# Parietal Alpha Asymmetry as a Correlate of Internet Use Severity in Healthy Adults

**DOI:** 10.3390/brainsci15111207

**Published:** 2025-11-08

**Authors:** Dovile Simkute, Povilas Tarailis, Inga Griskova-Bulanova

**Affiliations:** 1 Life Sciences Center, Institute of Biosciences, Vilnius University, Sauletekio ave 7, LT-10257 Vilnius, Lithuania; dovile.simkute@gmc.vu.lt (D.S.); povilas.tarailis@gmc.vu.lt (P.T.); 2 eBrain Lab, School of Mechatronic Systems Engineering, Simon Fraser University, Surrey, BC V3T 0A3, Canada; 3 Faculty of Medicine, Translational Health Research Institute, Vilnius University, LT-08406 Vilnius, Lithuania

**Keywords:** problematic internet use, electroencephalography, resting state, alpha asymmetry, alpha desynchronization, parietal asymmetry

## Abstract

**Background**: Problematic Internet Use (PIU) is associated with emotional and cognitive dysregulation, yet its neural correlates, particularly in non-clinical populations, remain poorly understood. This study investigated association of the resting-state alpha asymmetry and desynchronization with psychological correlates of internet use within healthy regular internet users. **Methods**: A total of 129 participants (49 males, aged 18–35) were assessed using the Nine-Item Problematic Internet Use Questionnaire (PIUQ 9), alongside measures of anxiety, depression, and obsessive–compulsive symptoms. Resting-state EEG was recorded across Eyes Open (EO) and Eyes Closed (EC) conditions, with frontal and parietal alpha asymmetry and desynchronization indices analyzed in relation to internet use severity (Spearman rank correlations with non-parametric bootstrapping, 5000 replicates; FDR-corrected). For further analysis, participants with the lowest (*n* = 36) and highest (*n* = 33) PIUQ-9 scores were classified as low and high PIU groups, respectively, and their neurophysiological profiles were compared (Mann–Whitney U tests). **Results**: Higher internet use severity was associated with greater right parietal alpha power during EO condition, indicating greater left hemisphere parietal activity among individuals with higher internet engagement. Individuals with higher internet use severity also exhibited reduced absolute frontal and parietal alpha power, while alpha desynchronization was not associated with PIU severity or psychological symptoms. **Conclusions**: These findings suggest that posterior asymmetry patterns may serve as a neurophysiological correlate of PIU in non-clinical populations, warranting further investigation in future research.

## 1. Introduction

The rapid expansion of internet technology has made digital interactions integral to daily life, blurring distinctions between online and offline worlds, particularly among younger demographics who report near-constant connectivity with digital platforms [1,2]. However, the psychological and behavioral consequences of internet use vary greatly depending on the nature and extent of individual engagement. While thoughtful and purposeful usage may foster personal development, knowledge acquisition, and social connectedness, excessive or maladaptive engagement can lead to significant psychosocial and functional impairments, broadly referred to as Problematic Internet Use (PIU) [3,4,5,6,7,8].

PIU, estimated to affect around 7% of the global population [9], is typically characterized by impaired control over internet use and significant negative outcomes, manifesting along a continuum that ranges from healthy to problematic involvement [4,5,10,11,12,13]. Core addiction-like symptoms are commonly reported in PIU [4,14,15,16], yet their expression and interpretation vary significantly across different online contexts, contributing to ongoing conceptual and diagnostic ambiguities [5,17,18,19,20,21,22]. Nonetheless, the pervasive role of internet use has prompted extensive exploration of PIU correlates, consistently revealing dynamic interactions among physical, psychological, social, cognitive, and neurophysiological factors [3,5,6,16]. Despite this growing body of work, significant gaps remain in understanding the mechanisms underlying the onset and maintenance of PIU.

A central challenge in addressing PIU lies in differentiating pathological behavior from high internet engagement which is not necessarily pathological [5,23]. Importantly, time spent online is not a reliable indicator of pathology; instead, the context of use and its consequences are critical factors [23,24,25]. The integration of internet use into daily life might mask the recognition of misuse and its warning signs, and problematic patterns can go unrecognized, both by users and their communities. Therefore, approaching PIU as a spectrum of engagement—ranging from healthy to excessive and problematic—offers a more nuanced perspective, enabling the identification of subthreshold, emerging, and acute manifestations, particularly within non-clinical populations [10,13]. However, despite this, a substantial portion of the literature continues to treat PIU as a binary condition (severely expressed vs. absent), which risks overlooking these spectrum-level nuances.

Neuroimaging studies, including EEG and fMRI, have documented characteristic alterations in brain activity during rest, suggesting their potential as candidate PIU biomarkers [26,27,28,29,30]. Particularly relevant might be the alpha activity (8–12 Hz), conceptualized as a mechanism for regulating the timing of neural inhibition and gating of information flow [31], contributing to inhibitory processes by facilitating the synchronization of large-scale neural brain networks [32,33]. The reduction in alpha power observed during eyes-open states compared to eyes-closed conditions, commonly referred to as alpha desynchronization, is generally interpreted as an index of increased arousal, reflecting a state in which large populations of neurons cease to oscillate synchronously during active information processing [34,35]. Furthermore, due to the inverse relationship between alpha power and cortical activity, greater alpha power in one hemisphere is interpreted as a sign of reduced neural activity in that region [32]. This hemispheric imbalance in alpha power is commonly examined through alpha asymmetry, defined as the relative difference in alpha power between homologous left and right brain regions [32,33]. Resting-state alpha parameters have been consistently associated with affective, motivational, and cognitive processes, with atypical patterns observed in various neuropsychiatric and neurodevelopmental conditions [36]. Some prior works identified its possible utility in PIU [29,37,38]; however, its potential in the context of PIU has not been fully addressed. Notably, while alpha asymmetry is a widely studied marker of brain lateralization, investigations have primarily focused on frontal regions, with parietal asymmetry receiving comparatively little attention despite its relevance to attention and sensory integration [39,40,41].

To address this gap, we aimed to investigate neurophysiological alpha activity-based markers (alpha power, alpha asymmetry, and alpha desynchronization) across frontal and parietal regions within a non-clinical population of internet users, thereby extending prior PIU work that has focused primarily on frontal measures. By relating these measures to continuous PIU severity and to high/low internet use contrasts, this spectrum-oriented approach targets subclinical manifestations between casual and disordered internet use and offers insights into neural patterns that may signal emerging dysfunction before substantial impairments develop. Given that regular internet users far outnumber problematic users, such approach extends a literature that has predominantly treated PIU dichotomously (present vs. absent).

## 2. Methods

### 2.1. Participants

A total of 161 participants (71 males and 90 females) with an age range of 18 to 35 years old (mean age of 24.21 ± 4.28 years) participated in the study. Participants had to be in good overall health; report no past or current psychiatric, neurological, or endocrine disorders; and have no psychoactive substance use or addictions; participant sampling, recruitment, and screening procedures are detailed in Simkute et al. [42]. All participants gave written informed consent after a full explanation of the procedures. The protocol was approved by the Vilnius Regional Biomedical Research Ethics Committee (Nr.2019/10-1159-649).

### 2.2. Questionnaires

The Nine-Item Problematic Internet Use Questionnaire (PIUQ-9) [43] has been established as a reliable tool for identifying the extent of internet misuse. It comprises nine items and retains the original three-factor structure of the 18-item Problematic Internet Usage Questionnaire [44]: obsession, neglect, and control disorder. The total score spans 9-45; a provisional cutoff of 22 flags Problematic Internet Use, with higher scores indicating greater internet use severity.

The Beck Anxiety Inventory (BAI) [45] is a widely used self-report measure for evaluating the severity of anxiety symptoms. The BAI comprises 21 items, each rated on a Likert-type scale from 0 (not at all) to 3 (severely) based on the individual’s reported experience of anxiety symptoms over the period of the last two weeks. Total scores can range from 0 to 63 points, with higher scores indicating greater severity of anxiety.

Beck’s Depression Inventory (BDI-II) [46] assesses depressive symptom severity and is widely used in both research and clinical contexts [47,48]. The inventory includes 21 items, each scored on a scale from 0 (completely disagree) to 3 (completely agree), based on the individual’s reported experience of depressive symptoms over the past two weeks, yielding a total score from 0 to 63, with higher scores indicating more severe depression.

The Clark–Beck Obsessive–Compulsive Inventory (CBOCI) [49] assesses the frequency and severity of obsessive and compulsive symptoms. It is a 25 item measure with two subscales—obsessions (14 items) and compulsions (11 items). Items are rated on four statements scored 0–3 (reflecting increasing frequency/severity), yielding a total score of 0 to 72, with higher scores indicating greater symptom severity.

### 2.3. EEG Recording and Processing

EEG acquisition and preprocessing followed standard procedures and are described in greater detail in our task-based ERP study [42]. Procedures specific to the present resting-state dataset are described herein.

Participants sat comfortably upright in a low-light, sound-attenuated, electrically shielded room. Resting-state EEG was acquired with a 64-channel WaveGuard cap (Ag/AgCl; international 10–10 montage) using ANT Neuro equipment (Hangelo, The Netherlands). Ocular activity was monitored with an electro-oculogram (EOG): vertical EOG from electrodes placed above and below the right eye, and horizontal EOG from electrodes positioned at the left and right outer canthi. All signals were referenced to the mastoids, with the ground near Fz, impedances were kept < 20 kΩ, and signals were digitized at 2048 Hz.

At the start of the resting-state EEG session, participants were instructed to keep their eyes open (EO condition) for the first two minutes of the session. Following an alarm indicating a change, they were asked to close their eyes (EC condition) for the next two minutes, while resting-state EEG signals were recorded. Research indicates that a 2 min EEG recording provides asymmetry scores with internal consistency and reliability comparable to an 8 min recording [50]. Throughout the session, participants were instructed to avoid movement and remain still, relax, and avoid engaging in specific thoughts. In the EO condition, they were directed to fixate their gaze on a white cross against a grey background on a screen placed in front, and not to fall asleep during the eyes-closed condition. Throughout the session, EEG recordings for all participants were actively monitored to ensure compliance with instructions.

The offline EEG data processing was performed in MATLAB (The Mathworks, Natick, MA, USA) using the EEGLAB toolbox (version 2022.0) [51]. The 50 Hz power line noise was attenuated using the CleanLine plugin for EEGLAB, after which independent component analysis (ICA) analysis (‘runica’, default settings) was used to identify and remove ocular and cardiac components. Records were then visually inspected; channels with exhibiting excessive noise were excluded and reconstructed via spherical spline interpolation [52]. Data were band-pass-filtered (0.1–30 Hz; second-order Butterworth), re-referenced to the average, and segmented into non-overlapping, artifact-free 2 s epochs with baseline adjustment across each segment. On average, EO yielded 53.86 ± 5.13 artifact-free epochs and EC 53.76 ± 6.16; participants were retained only if ≥40 clean epochs were available.

### 2.4. Alpha Power, Alpha Asymmetry, and Alpha Desynchronization Assessment

Power spectra (μV2) were derived from EEG data by applying MATLAB’s Fast Fourier Transform (FFT) function using a non-overlapping Hanning window (FieldTrip parameters mtmfft and Hanning). The mean frontal and parietal alpha power were calculated by averaging absolute power over predefined alpha frequencies (8–12 Hz) across respective electrodes (frontal: F7, F3, Fz, F4, F8, and parietal: P7, P3, Pz, P4, P8) and conditions (eyes open (EO) and eyes closed (EC)) for each participant.

Alpha asymmetry index (*AAI*) was computed as the log-transformed difference between right and left alpha power values [32,33]:

$$AAI=\ln\left(R)-ln(L\right)$$

where L and R denote left (F3, F7, P3 or P7) and right (F4, F8, P4 or P8) electrode site responses, respectively. The FFT-derived alpha power was analyzed at four paired electrode sites to assess middle (F3/F4 and P3/P4) and lateral (F7/F8 and P7/P8) alpha asymmetry. Alpha asymmetry scores were calculated for each condition (EO, EC), with positive scores indicating greater right alpha power (and greater relative left hemisphere activity) and negative scores indicating greater left alpha power (and relative right hemisphere activity).

Alpha desynchronization index (*D*) was determined as the difference in the average alpha power between the eyes-open (EO) and eyes-closed (EC) conditions, and calculated as

D=EO−EC

across 10 electrode sites (F3, F4, Fz, F7, F8, P3, P4, Pz, P7, P8) for each participant.

### 2.5. Statistical Analysis

Two complementary sample evaluation strategies were implemented. Continuous associations (1): across the full sample (*n* = 129), relations among PIUQ-9, psychological measures (BAI, BDI-II, CBOCI), and EEG indices (alpha power, alpha asymmetry, alpha desynchronization) were quantified using Spearman rank correlations. Distributional assumptions were screened with Shapiro–Wilk tests; robustness was enhanced via non-parametric bootstrapping (5000 replicates), significance threshold was set at α = 0.05, and *p* values underwent False Discovery Rate (FDR) [53]-based multiple-comparison adjustment.

Group-based approach (2): Participants were classified in the bottom and top PIUQ-9 score quartiles (inclusive thresholds) as Low PIU (scores 10–16; *n* = 36) and High PIU (scores 23–36; *n* = 33). The lower bound of 23 closely aligns with the provisional cutoff score of 22 [43], supporting this approach in a non-clinical cohort. Group differences in alpha power, asymmetry, and desynchronization were evaluated with Mann–Whitney U tests following Shapiro–Wilk and Levene’s tests indicating violations of normality and homogeneity of variance. Non-parametric bootstrapping (5000 replicates) and FDR correction were applied.

Tables report raw *p*-values; effects surviving FDR correction (α = 0.05) are flagged with an asterisk, with FDR-adjusted *p*-values provided in text.

Statistical analyses were conducted using MS Excel (version 2053, Microsoft Corporation, Redmond, Washington, United States, 2018), JASP 0.18.3 (JASP Team, Amsterdam, Netherlands, 2024), and SPSS (version 29.0.2.0, IBM SPSS, Armonk, New York, United States). Statistical power was evaluated in G*Power (version 3.1.9.4). For the correlation analyses (n = 129, two-tailed α = 0.05 and 80% power), the minimal detectable effect was r = 0.245; an effect of r = 0.30 yields power = 0.94. For high (n = 33) and low (n = 36) PIU group comparisons (Wilcoxon–Mann–Whitney; two-tailed α = 0.05, power = 0.80), the minimal detectable effect was d = 0.70, corresponding to rank-biserial r = 0.38.

## 3. Results

Out of 161 enrolled participants, 31 datasets were excluded (technical issues: n = 12, poor recording quality: n = 7, outliers: n = 6), corresponding to 19.3% attrition and yielding a final sample of 129 (49 males, 80 females; age = 24 ± 4.02 years). The PIUQ-9 mean for the full sample was 19.39 ± 5.2 (range 10–36), below the provisional cutoff of 22 of PIU [43], although individual scores spanned from low to elevated severity.

Psychological questionnaires indicated a BAI group mean in the moderate range of anxiety, with a subset exceeding common clinical thresholds. The BDI-II scores were low on average but showed notable interindividual variability in depressive symptoms; CBOCI totals reflected heterogeneity in obsessive–compulsive traits, with obsession scores generally exceeding compulsion scores. Collectively, the current sample exhibits varying levels of psychological symptoms. Descriptive statistics are provided in Table 1.

Descriptive statistics for internet usage patterns and psychological measures within the sample are provided in Table 1.

### 3.1. Correlations Between Internet Use Severity and Psychological Measures

As anticipated, higher PIUQ-9 scores correlated with poorer psychological profiles, closely mirroring findings from our prior report on the same cohort [42]. PIUQ-9 correlated positively with all measures assessed—anxiety, depression, obsessions, compulsions, and total obsessive–compulsive symptoms (BAI: rs = 0.316, *p* < 0.001, FDR-corrected *p* < 0.001; BDI: rs = 0.285, *p* < 0.001, FDR-corrected *p* = 0.002; CBOCI obsessions: rs = 0.355, *p* < 0.001, FDR-corrected *p* < 0.001; CBOCI compulsions: rs = 0.349, *p* < 0.001, FDR-corrected *p* < 0.001; total CBOCI score: rs = 0.406, and *p* < 0.001, FDR-corrected *p* < 0.001). Detailed results are provided in the Appendix A Table A1.

### 3.2. Correlations Between Internet Use Severity and Alpha Power

**Figure 1:**
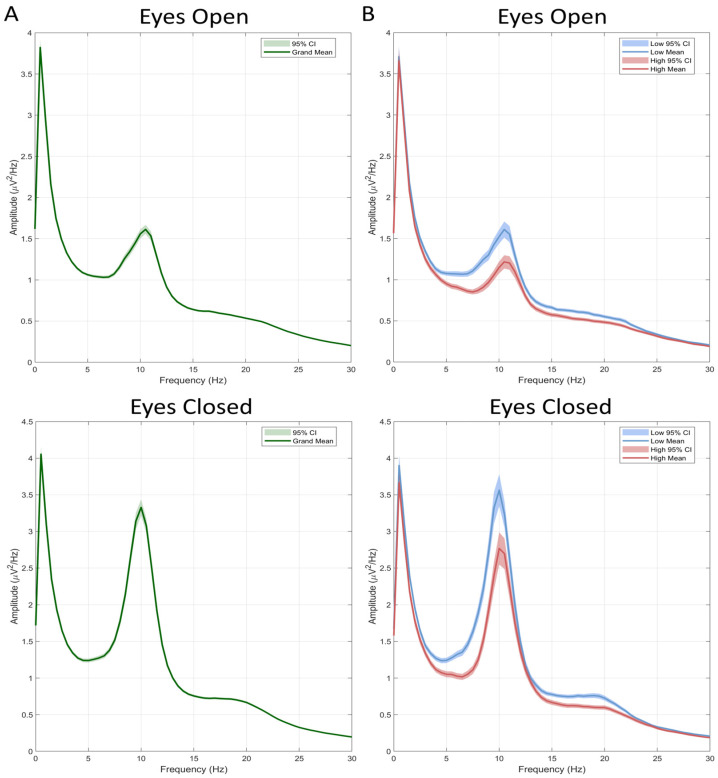
Absolute power grand-average across the full sample (n = 129) (**A**) and separately in the High (n = 33) and Low (n = 36) PIU groups (**B**). Shaded regions indicate the 95% confidence interval for the mean power.

**Figure A1:**
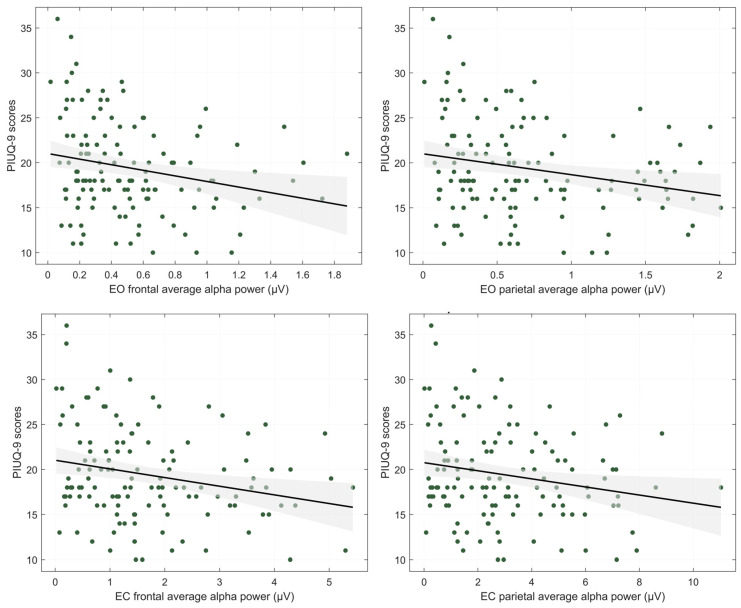
Correlation plots of internet use severity (PIUQ-9 scores) with average absolute alpha power (μV) in frontal and parietal regions. EO—eyes-open condition, EC—eyes-closed condition. Shaded region indicates the 95% confidence interval of the regression estimates.

The absolute power grand-averages across all participants during eyes-open (EO) and eyes-closed (EC) conditions are presented in Figure 1A. Descriptive statistics for absolute alpha power averaged within frontal and parietal regions are provided in Table 2. Significant negative correlations were observed between PIUQ-9 scores and alpha power at frontal (rs = −0.248, *p* = 0.005, FDR-corrected *p* = 0.015) and parietal (rs = 0.209, *p* = 0.018, FDR-corrected *p* = 0.022) regions during EO, and frontal (rs = −0.241, *p* = 0.006, FDR-corrected *p* = 0.015) and parietal (rs = −0.183, *p* = 0.038, FDR-corrected *p* = 0.038) regions during EC, but not with BAI, BDI or CBOCI scores. Correlation results and corresponding plots are provided in the Appendix A (Table A2 and Figure A1, respectively).

### 3.3. Correlation Between Internet Use Severity and Alpha Asymmetry Index

**Figure 2:**
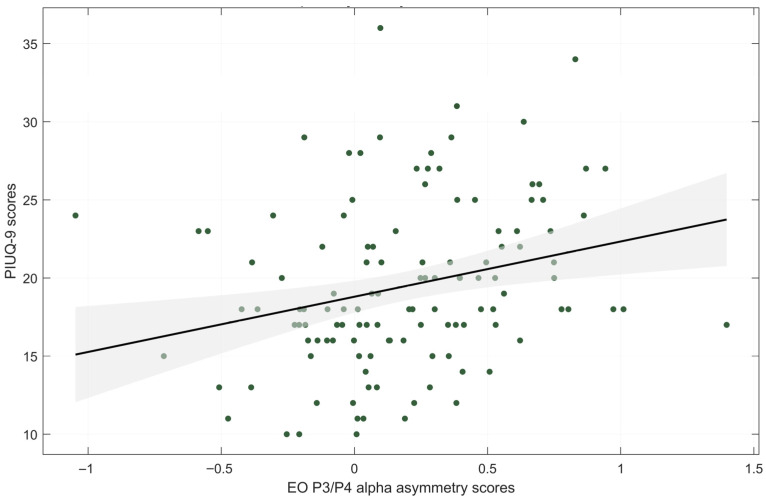
Correlation plot of PIU severity (PIUQ-9 scores) with alpha asymmetry scores middle parietal sites. EO—eyes-open condition. Shaded region indicates the 95% confidence interval of the regression estimates.

Descriptive statistics for alpha asymmetry scores during EO and EC conditions are presented in Table 2. A significant positive correlation was observed between PIUQ-9 scores and alpha asymmetry score at P3/P4 during the EO condition (rs = 0.317, *p* < 0.001, FDR-corrected *p* = 0.003) (Figure 2), indicating greater right parietal alpha power—and thus increased left hemisphere activity—with higher levels of internet use. Detailed statistical results are presented in Table 3, and correlations between asymmetry scores and psychological variables are provided in Appendix A Table A3.

### 3.4. Correlations Between Internet Use Severity and Alpha Desynchronization Scores

Although several initial associations were observed between internet use and alpha desynchronization scores, none remained significant after FDR correction. Additionally, no significant correlations were found between alpha desynchronization scores and psychological variables. Detailed statistical results are presented in Appendix A Table A4. Descriptive statistics for alpha desynchronization scores during EO and EC conditions are presented in Appendix A Table A5.

### 3.5. Low vs. High Internet Use Engagement

The Low (*n* = 36; 22 females; PIUQ-9 score 13.72 ± 2.07, range 10–16) and High PIU (*n* = 33, 24 females; PIUQ-9 score 26.49 ± 3.1, range 23–36) groups did not differ in age (*p* = 0.329). Groups diverged on all psychological measures (after FDR correction all *p* < 0.001), with the exception of BDI (also significant, FDR-corrected *p* = 0.018) (Table 4). Descriptive statistics are provided in Appendix A Table A6.

Comparisons of alpha power between High and Low PIU groups revealed significant differences across both EO and EC conditions over frontal (EO: *p* = 0.008, FDR-corrected *p* = 0.018; EC: *p* = 0.008, FDR-corrected *p* = 0.018) and parietal (EO: *p* = 0.046, FDR-corrected *p* = 0.049; EC: *p* = 0.049, FDR-corrected *p* = 0.049) regions. Detailed results are displayed in Table 5 and Figure 1B, with descriptive statistics provided in the Appendix A Table A6.

**Figure 3:**
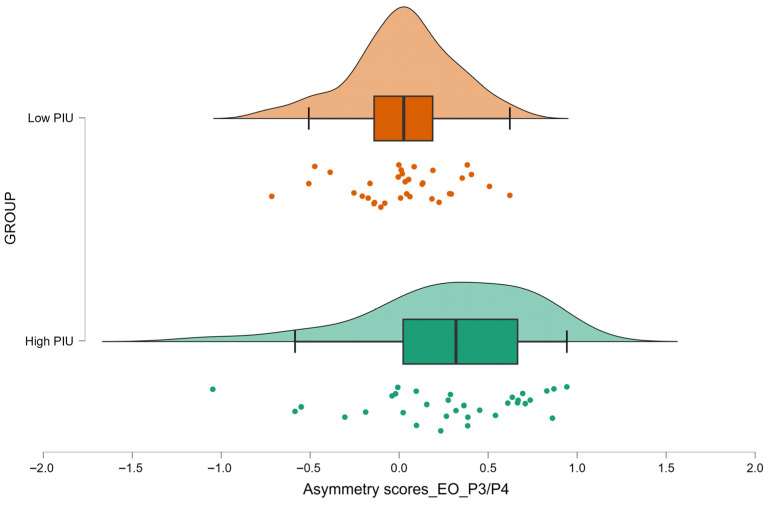
Raincloud plot (kernel density estimates, boxplots, and individual data points) of parietal asymmetry scores during eyes-open (EO) condition at P3/P4 site for individuals classified as High PIU and Low PIU. Individual dots represent the asymmetry scores for each participant.

Consistent with the whole-sample correlations, the High PIU group showed higher alpha asymmetry values at P3/P4 during the EO condition than the Low PIU group (*p* = 0.002, FDR-corrected *p* = 0.024; 0.28 ± 0.46 vs. 0.02 ± 0.29, respectively) (Figure 3). Detailed statistics are provided in Table 6, with descriptive values in Appendix A Table A6.

No significant differences between the groups were found for alpha desynchronization scores; detailed results provided in Appendix A Table A7. Descriptive statistics are presented in Appendix A Table A6.

## 4. Discussion

The present study examined EEG resting-state alpha power, asymmetry, and desynchronization in relation to internet use severity, focusing on frontal and parietal regions in a sample of 129 healthy adults. Results revealed a significant association between internet use severity and alpha asymmetry at the P3/P4 site during the eyes-open condition when measured in the whole sample and compared between High and Low PIU groups. Specifically, higher PIUQ-9 scores were linked to greater left parietal activation during the eyes-open condition, and with lower absolute alpha power in both eyes-open and eyes-closed conditions. Moreover, no association emerged between alpha desynchronization and internet use or any of the psychological variables assessed (symptoms of depression, anxiety, obsessions–compulsions).

These findings align with a broader body of literature suggesting that PIU and internet-related disorders are associated with altered resting-state brain activity within frontal and parietal regions, with alterations significantly correlating with PIU severity [26,28,54]. In line with current findings, reduced frontal and parietal alpha power was observed in excessive gamers [55], and PIU with comorbid depression [27]; however, the latter finding implies that a decrease in global alpha power, even though commonly observed, is not specific to PIU.

Although significant associations between internet use severity and alpha power were found in the current study, no such relationship was observed for alpha desynchronization. Alpha desynchronization refers to the suppression of alpha activity, typically observed during transitions from a resting state (e.g., eyes closed) to an active state (e.g., eyes open or during sensory stimulation). This phenomenon, characterized by a reduction in alpha power and connectivity, reflects increased cortical activation and engagement of task-relevant neural regions [56,57,58]. The effect is most pronounced posteriorly, and is associated with heightened visual system activity and widespread thalamo-cortical communication, activating the entire cortex and indicating readiness for information processing [56]. By contrast, alpha synchronization is often interpreted as a top-down inhibitory mechanism that gates irrelevant input [35], whereas desynchronization reflects a transition to a more irregular, low-voltage state supporting stimulus processing [59]. In the context of PIU, prior work has reported reduced task-evoked frontal alpha/beta desynchronization during stop-signal tasks and negative links between Internet Addiction Test scores and resting frontal desynchronization [60]. In the present sample, however, alpha desynchronization showed no relation to internet-use severity.

The significant finding concerning alpha asymmetry warrants further discussion. A general theory posits that resting-state alpha asymmetry reflects trait-like predispositions toward affective (positive vs. negative) and motivational (approach vs. avoidance) orientations. According to the approach–withdrawal model [61], greater relative left frontal activity is linked to approach and positive affect, whereas right frontal dominance relates to withdrawal and negative affect [32,33,62,63]. Despite extensive work on frontal asymmetry, parietal alpha asymmetry remains comparatively underexplored [40,41], even though the parietal cortex is central to attention allocation and polymodal integration [39]. Within the valence–arousal model [64], higher left frontal activity has been associated with anxious apprehension (i.e., worry), whereas right parietal activity reflects anxious arousal (e.g., panic) [64,65,66]. Furthermore, parietal alpha asymmetry has also been associated with individual personality differences, emotion processing and emotional vulnerability, dysregulated behavioral activation, depression, anhedonia, and traits like rumination and self-criticism [40,66,67]. Notably, parietal asymmetry often exhibits patterns opposite to frontal asymmetry [40,67]. For instance, depressive symptoms, emotional vulnerability, internalizing symptoms, and greater behavioral activation (BAS) have been attributed to left-lateralized parietal activity, contrasting with right-lateralized frontal effects [40,67,68,69].

Furthermore, a review by Burleigh et al. [26] summarized increased right hemisphere alpha coherence (including parietal regions) in gamers, probably associated with consistent activation of visuospatial working memory and executive function in frequent gaming. In support, individuals with PIU exhibit reduced functional connectivity in the left parietal lobe of the right frontoparietal network [28], suggesting impaired cross-hemispheric network communication. Consistent right frontal activity has also been observed in Problematic Social Media users [37] and Internet Addiction (IA) groups [38], with this right-dominant frontal pattern linked to poorer emotion regulation choices in IA. To our knowledge, parietal alpha asymmetry has not been investigated within the PIU field. The present finding suggesting greater relative left-lateralized parietal activity among healthy individuals with more severe internet engagement might align with these findings, albeit speculatively, considering that parietal asymmetry often inverts frontal patterns [40,67]. Moreover, the current findings generally do not contradict but rather extend the results of a pilot study on recreational internet users by G. Y. Wang & Griskova-Bulanova [29]; also, no association between internet use and frontal alpha asymmetry was detected in the present larger sample, while identifying a parietal effect that was not assessed previously.

Notably, internet use severity was associated with greater left parietal activity only in the eyes-open (EO) condition, but not during the eyes-closed (EC) condition. This discrepancy likely reflects distinct neurophysiological baselines [56,70]: EC entails reduced sensory input and stronger thalamo-cortical rhythmicity (a lower arousal state), making it less sensitive to individual differences in attention or motivation, whereas EO—even under minimal visual stimulation—elicits alpha desynchronization and widespread cortical activation, including frontoparietal networks involved in attention control [56,70]. Moreover, brain regions associated with unconstrained mental activities (such as self-reflective thought, environmental monitoring, and mind wandering) may be differentially engaged under different resting-state conditions [71], and default mode network (DMN) regions involved in sensory monitoring and salience evaluation exhibit higher functional connectivity in EO than EC [72]. Consistent with this, IA induces widespread large-scale brain network alterations rather than isolated regional changes, with disruptions including DMN and reward pathways—pointing to shifts in reward valuation, impulsivity, salience attribution, and cue reactivity [72]—patterns that further justify the greater sensitivity of EO measures.

Furthermore, alpha asymmetry has been proposed as a neurophysiological index of both psychological traits and underlying regulatory mechanisms [33]. Parietal asymmetry, in particular, has been associated with behavioral approach/inhibition systems; greater left-sided parietal activity has been linked to higher behavioral activation [67], a profile that may characterize individuals with more compulsive internet engagement. Additionally, frontal and parietal asymmetries relate to attentional bias independent of subclinical mood or anxiety variation [73], suggesting that such asymmetry indexes underlie top-down attentional control tendencies and trait-like attentional biases rather than psychopathology per se. In line with this, heavy media multitaskers show heightened susceptibility to distraction, reduced filtering of irrelevant input, and a greater reliance on bottom-up, exploratory control relative to light multitaskers [74]. Such responsivity is expected to be more prominent during EO, when attentional systems are in a more reactive, externally focused state. Together with findings of reduced global alpha power in higher internet users (corresponding to increased cortical excitability and lower perceptual thresholds [75]), this interpretation aligns with theoretical PIU models emphasizing compulsive, stimulus-driven engagement and weakened top-down attentional regulation [4,76,77]. Taken together, EO and EC constitute qualitatively distinct conditions and highlight EO as a more sensitive context for detecting PIU-related asymmetry patterns, particularly those linked to attentional dysregulation in regular internet users.

Moreover, the current results did not identify an association between depressive symptoms and alpha asymmetry. Although alpha asymmetry has been widely examined in depression, findings are inconsistent and the direction of effects remains unresolved [78]. Some reports link left-lateralized hypoactivation and greater right frontal/parietal activity to depressive symptoms [29,32,79,80]. Notably, although in the current sample PIU and depression were correlated (rs = 0.285, *p* = 0.001), the initially observed negative association between depression symptoms and parietal asymmetry did not survive FDR correction; only PIU retained a positive association with parietal alpha asymmetry. This pattern tentatively points to divergent lateralization: a trend towards greater right parietal activity in association with depression (in line with prior research) versus greater left parietal activity in relation to PIU. This may suggest distinct lateralized neural processes underlying PIU and depressive symptoms, despite their overlapping psychological features within a non-clinical sample of regular internet users.

The present study advances the field of PIU research by identifying parietal alpha asymmetry as a potential specific correlate for internet use severity. This result is notable given that posterior alpha activity is maximal during rest and shows greater temporal stability and consistency across conditions than frontal asymmetry [40,66,81]. Our findings suggest that asymmetry-based indices may be more specific to the neural characteristics underlying PIU, whereas desynchronization—reflecting general cortical arousal—may not capture these vulnerabilities, as no associations were observed. Investigating parietal alpha asymmetry within PIU offers a novel yet theoretically grounded approach to investigate alterations and/or dysfunctions of neural mechanisms within increasing internet use, addressing a clear gap in the current literature.

## 5. Limitations

While alpha asymmetry is a well-established neurophysiological measure, it is not considered a sufficient standalone diagnostic biomarker; rather, it may serve as a meaningful index of symptom expression, risk status, or treatment response [32,36]. In the resting state, the interpretability of alpha asymmetry is constrained by spontaneous, internally driven cognitive processes that fluctuate across individuals and time and may modulate both affective and cognitive components [32]. Whether the alpha power/asymmetry pattern observed here reflects a pre-existing risk factor or a consequence of excessive internet use remains unresolved and warrants future investigation. Furthermore, the present study was restricted to scalp-level EEG alpha power and alpha asymmetry derived from predefined frontal and parietal electrode pairs; network-level metrics (e.g., functional connectivity or connectivity asymmetry) were not assessed. Future studies should test whether connectivity provides explanatory value beyond the scalp-level alpha indices in non-clinical samples of internet users.

## 6. Conclusions

As PIU unfolds on a continuum of maladaptive online engagement, brief resting-EEG markers may help characterize spectrum-level variation in internet use severity within non-clinical populations. Addressing a gap beyond frontal alpha indices, the present findings from a sample of 129 healthy young adult regular internet users extend prior work on frontal asymmetry by demonstrating that parietal asymmetry may also serve as a meaningful neural index for problematic behaviors, such as PIU. This study showed that greater internet use severity is associated with distinct patterns in resting-state alpha activity, specifically increased left hemisphere activity at parietal sites. Additionally, individuals with higher PIU scores exhibited globally reduced alpha power, indicative of decreased cortical inhibition, while alpha desynchronization did not differentiate between levels of internet use severity or psychopathological symptoms. Furthermore, measured neurophysiological correlates did not reveal associations with any of the psychopathological variables assessed. Collectively, these findings highlight the potential relevance of lateralized parietal activity as a neurophysiological correlate of internet use severity, probably reflecting alterations in attentional control mechanisms. However, subsequent studies should confirm the effects.

## Figures and Tables

**Table 1:** Descriptive statistics for the internet usage patterns and psychological characteristics within the sample.

Variable	Valid (*n*)	Mean	SD	Range (Min–Max)
PIUQ-9	129	19.39	5.2	10–36
BAI	128	31.78	7.39	21–57
BDI	125	10.37	8.77	0–48
CBOCI	128	19.59	11.72	0–56
CBOCI_obsessions	128	11.84	6.73	0–33
CBOCI_compulsions	129	7.8	6.12	0–30

Sample size differences reflect missing or incomplete responses on the psychological measures. BAI—Beck Anxiety Inventory; BDI—Beck Depression Inventory; CBOCI—Clark–Beck Obsessive–Compulsive Inventory, PIUQ-9—The Nine-Item Problematic Internet Use Questionnaire.

**Table 2:** Descriptive statistics for averaged alpha power (μV2) and alpha asymmetry scores within frontal and parietal regions during eyes-open (EO) and eyes-closed (EC) conditions in the full sample.

Variable	*n*	Mean ± SD	Variable	*n*	Mean ± SD	Variable	*n*	Mean ± SD
Alpha frequency (μV2)			Alpha asymmetry (EO)			Alpha asymmetry (EC)		
EO_frontal	128	0.52 ± 0.39	F3/F4	125	−0.03 ± 0.24	F3/F4	128	−0.05 ± 0.19
EO_parietal	128	0.72 ± 0.58	F7/F8	124	−0.05 ± 0.50	F7/F8	128	−0.06 ± 0.43
EC_frontal	129	1.71 ± 1.30	P3/P4	122	0.19 ± 0.40	P3/P4	123	0.33 ± 0.48
EC_parietal	129	3.05 ± 2.34	P7/P8	120	0.13 ± 0.41	P7/P8	121	0.33 ± 0.55

Varying sample sizes reflect pairwise deletion due to condition- or channel-specific data quality.

**Table 3:** Spearman’s Correlation coefficients and *p* values for internet use (PIUQ-9 scores) and alpha asymmetry for all electrode pairs (F3/F4, F7/F8, P3/P4, P7/P8) during eyes-open and eyes-closed conditions.

Variable		Eyes Open	Eyes Closed
F3/F4	Spearman’s rho	0.096	−0.038
	*p*-value	0.286	0.668
	*n*	125	128
F7/F8	Spearman’s rho	0.019	−0.022
	*p*-value	0.834	0.805
	*n*	124	128
P3/P4	Spearman’s rho	0.317 *	0.128
	*p*-value	<0.001	0.159
	*n*	122	123
P7/P8	Spearman’s rho	0.19	0.176
	*p*-value	0.038	0.053
	*n*	120	121

Provided *p*-values are unadjusted; asterisks mark findings that remain significant after FDR correction.

**Table 4:** Group differences in psychological measures.

Variable	U	*p*	Effect Size	SE Effect Size
PIUQ-9	1188	<0.001 *	1	0.139
BAI	860	<0.001 *	0.489	0.14
BDI	748.5	0.018 *	0.337	0.141
CBOCI	923.5	<0.001 *	0.555	0.139
CBOCI obsessions	882	<0.001 *	0.485	0.139
CBOCI compulsions	893.5	<0.001 *	0.504	0.139

Provided *p*-values are unadjusted; asterisks mark findings that remain significant after FDR correction. Mann–Whitney U test. BAI—Beck Anxiety Inventory; BDI—Beck Depression Inventory; CBOCI—Clark–Beck Obsessive–Compulsive Inventory; PIUQ-9—The Nine-Item Problematic Internet Use Questionnaire; SE effect size—standard error of effect size; and U—Mann–Whitney U test statistic.

**Table 5:** Group differences for alpha power averaged for frontal and parietal regions during eyes-closed and eyes-open conditions.

Variable	U	*p*	Effect Size	SE Effect Size
EO_frontal	376	0.008 *	−0.367	0.139
EO_parietal	428	0.046 *	−0.279	0.139
EC_frontal	373	0.008 *	−0.372	0.139
EC_parietal	430	0.049 *	−0.276	0.139

Provided *p*-values are unadjusted; asterisks mark findings that remain significant after FDR correction. SE effect size—standard error of effect size; and U—Mann–Whitney U test statistic.

**Table 6:** Group differences between alpha asymmetry scores for all electrode pairs (F3/F4, F7/F8, P3/P4, P7/P8).

Variable	U	*p*	Effect Size	SE Effect Size
**Eyes Open**				
F3/F4	674	0.241	0.167	0.14
F7/F8	534	0.6	−0.075	0.14
P3/P4	801	0.002 *	0.428	0.141
P7/P8	704	0.021	0.333	0.143
**Eyes Closed**				
F3/F4	575	0.825	−0.032	0.139
F7/F8	524	0.406	−0.118	0.139
P3/P4	644	0.409	0.118	0.14
P7/P8	640	0.144	0.212	0.143

Provided *p*-values are unadjusted; asterisks mark findings that remain significant after FDR cor-rection. Mann–Whitney U test.

## Data Availability

The data presented in this study is available on request from the corresponding author. The data underlying this article are not publicly shared due to institutional and legal restrictions. Access to the data may be granted upon reasonable request to the corresponding author, pending approval from the relevant ethics and data protection authorities.

## Abbreviations

The following abbreviations are used in this manuscript:

EEG	Electroencephalography
EC	Eyes-closed
EO	Eyes-open
FDR	False Discovery Rate
PIU	Problematic Internet Use

## Appendix A

**Table A1:** Spearman’s correlations between psychological evaluation measures and scores of the Problematic Internet Use Questionnaire (PIUQ-9).

Variable		BAI	BDI	CBOCI	CBOCI Obsessions	CBOCI Compulsions
PIUQ-9	Spearman’s rho	0.316 *	0.285 *	0.406 *	0.355 *	0.349 *
	*p*-value	<0.001	0.001	<0.001	<0.001	<0.001
	n	128	125	128	128	129

Provided *p*-values are unadjusted; asterisks mark findings that remain significant after FDR correction. PIUQ-9—The Nine-Item Problematic Internet Use Questionnaire, BAI—The Beck Anxiety Inventory, BDI—Beck’s Depression Inventory, CBOCI—The Clark–Beck Obsessive–Compulsive Inventory.

**Table A2:** Spearman’s correlations between the scores of the Problematic Internet Use Questionnaire (PIUQ-9), psychopathological measures and averaged absolute alpha power within frontal and parietal regions during eyes-open and eyes-closed conditions.

Variable		PIUQ-9	BAI	BDI	CBOCI	CBOCI Obsessions	CBOCI Compulsions
EO frontal	Spearman’s rho	−0.248 *	−0.129	−0.118	−0.067	−0.108	−0.016
	*p*-value	0.005	0.15	0.193	0.452	0.227	0.857
	n	128	127	124	127	127	128
EO parietal	Spearman’s rho	−0.209 *	−0.095	−0.107	−0.042	−0.098	0.042
	*p*-value	0.018	0.288	0.235	0.637	0.275	0.639
	n	128	127	124	127	127	128
EC frontal	Spearman’s rho	−0.241 *	0.03	−0.004	−0.023	−0.092	0.067
	*p*-value	0.006	0.735	0.966	0.8	0.303	0.448
	n	129	128	125	128	128	129
EC parietal	Spearman’s rho	−0.183 *	0.027	−0.006	−0.023	−0.083	0.064
	*p*-value	0.038	0.763	0.943	0.801	0.351	0.474
	n	129	128	125	128	128	129

Provided *p*-values are unadjusted; asterisks mark findings that remain significant after FDR correction. PIUQ-9—The Nine-Item Problematic Internet Use Questionnaire, BAI—The Beck Anxiety Inventory, BDI—Beck’s Depression Inventory, CBOCI—The Clark–Beck Obsessive–Compulsive Inventory.

**Table A3:** Spearman’s correlations between psychopathological measures and alpha desynchronization (EO-EC) scores for all electrode sites.

Variable		BAI	BDI	CBOCI	CBOCI Obsessions	CBOCI Compulsions
**Eyes Open**						
F3/F4	Spearman’s rho	0.156	0.088	0.048	0.093	0.004
	*p*-value	0.084	0.334	0.598	0.302	0.965
	n	124	122	124	124	125
F7/F8	Spearman’s rho	0.035	0.059	0.017	0.008	0.015
	*p*-value	0.701	0.522	0.849	0.927	0.869
	n	123	121	123	123	124
P3/P3	Spearman’s rho	0.07	0.088	0.074	0.074	0.047
	*p*-value	0.446	0.342	0.417	0.417	0.609
	n	121	119	121	121	122
P7/P8	Spearman’s rho	−0.032	−0.199	−0.002	−0.04	0.034
	*p*-value	0.731	0.032	0.983	0.667	0.716
	n	119	117	119	119	120
**Eyes Closed**						
F3/F4	Spearman’s rho	0.021	0.04	−0.009	0.023	−0.017
	*p*-value	0.818	0.662	0.916	0.799	0.852
	n	127	124	127	127	128
F7/F8	Spearman’s rho	−22.61	−0.022	−0.042	0.01	−0.106
	*p*-value	1	0.813	0.638	0.907	0.235
	n	127	124	127	127	128
P3/P3	Spearman’s rho	−0.039	0.044	−0.027	−0.034	−0.01
	*p*-value	0.668	0.636	0.764	0.706	0.91
	n	122	119	122	122	123
P7/P8	Spearman’s rho	−0.013	−0.115	0.099	0.043	0.137
	*p*-value	0.89	0.218	0.28	0.642	0.135
	n	120	117	120	120	121

Provided *p*-values are unadjusted; no results survived FDR correction. BAI—The Beck Anxiety Inventory, BDI—Beck’s Depression Inventory, CBOCI—The Clark–Beck Obsessive–Compulsive Inventory.

**Table A4:** Spearman’s correlations between internet use (PIUQ-9) and alpha desynchronization (EO-EC) scores for all electrode sites.

Variable		Frontal Electrode Sites	Parietal Electrode Sites
F7/P7	Spearman’s rho	0.191	0.212
	*p*-value	0.034	0.02
	n	124	120
F3/F3	Spearman’s rho	0.15	0.195
	*p*-value	0.096	0.033
	n	125	120
Fz/Pz	Spearman’s rho	0.151	0.155
	*p*-value	0.09	0.086
	n	127	123
F4/P4	Spearman’s rho	0.167	0.139
	*p*-value	0.06	0.126
	n	127	122
F8/P8	Spearman’s rho	0.193	0.095
	*p*-value	0.031	0.303
	n	125	120

**Table A5:** Descriptive statistics for alpha desynchronization scores in the full sample (n = 129).

Variable	Mean	SD
F7	−0.97	0.92
F3	−1.26	1.23
Fz	−1.42	1.40
F4	−1.20	1.17
F8	−0.93	0.90
P7	−2.07	1.93
P3	−1.42	1.39
Pz	−1.52	1.59
P4	−2.26	2.34
P8	−3.36	3.39

**Table A6:** Descriptive statistics for psychological measures, alpha power, alpha asymmetry and alpha desynchronization scores between High (n = 33) and Low (n = 36) internet use groups.

Variable	Group	Mean	SD
**Psychopathological measures**			
PIUQ-9	High PIU	26.49	3.14
	Low PIU	13.72	2.07
BAI	High PIU	36.03	8.66
	Low PIU	29.06	5.64
BDI	High PIU	13.59	10.20
	Low PIU	7.66	5.94
BOCI	High PIU	26.88	13.03
	Low PIU	14.97	9.09
CBOCI obsessions	High PIU	15.55	6.88
	Low PIU	9.72	5.56
CBOCI compulsions	High PIU	11.33	7.37
	Low PIU	5.36	4.51
**Alpha frequency (μV)**			
EO_frontal	High PIU	0.39	0.33
	Low PIU	0.61	0.4
EO_parietal	High PIU	0.54	0.47
	Low PIU	0.81	0.57
EC_frontal	High PIU	1.32	1.18
	Low PIU	2.04	1.31
EC_parietal	High PIU	2.51	2.21
	Low PIU	3.52	2.24
**Alpha asymmetry scores**			
**Eyes Open**			
F3/F4	High PIU	−0.01	0.25
	Low PIU	−0.06	0.24
F7/F8	High PIU	−0.13	0.69
	Low PIU	−0.04	0.38
P3/P4	High PIU	0.28	0.46
	Low PIU	0.02	0.29
P7/P8	High PIU	0.29	0.51
	Low PIU	0.04	0.35
**Eyes closed**			
F3/F4	High PIU	−0.08	0.22
	Low PIU	−0.05	0.16
F7/F8	High PIU	−0.14	0.63
	Low PIU	−0.02	0.28
P3/P4	High PIU	0.41	0.55
	Low PIU	0.30	0.49
P7/P8	High PIU	0.49	0.70
	Low PIU	0.27	0.40
**Alpha desynchronization scores (EO-EC)**			
F7	High PIU	−0.80	0.81
	Low PIU	−1.24	1.07
F3	High PIU	−1.00	0.96
	Low PIU	−1.49	1.32
Fz	High PIU	−1.15	1.20
	Low PIU	−1.67	1.48
F4	High PIU	−0.96	1.03
	Low PIU	−1.46	1.25
F8	High PIU	−0.77	0.84
	Low PIU	−1.18	1.01
P7	High PIU	−1.73	1.98
	Low PIU	−2.72	2.18
P3	High PIU	−1.14	1.22
	Low PIU	−1.85	1.73
Pz	High PIU	−1.34	1.59
	Low PIU	−1.68	1.50
P4	High PIU	−2.04	2.23
	Low PIU	−2.64	2.17
P8	High PIU	−3.07	3.26
	Low PIU	−3.88	3.27

BAI—Beck Anxiety Inventory; BDI—Beck Depression Inventory; CBOCI—Clark–Beck Obsessive–Compulsive Inventory; PIUQ-9—The Nine-Item Problematic Internet Use Questionnaire.

**Table A7:** Group differences between alpha desynchronization (EO-EC) scores for all electrode sites.

Variable	U	*p*	Effect Size	SE Effect Size
F7	734	0.055	0.271	0.14
F3	705	0.12	0.221	0.14
Fz	710	0.106	0.229	0.14
F4	753	0.057	0.268	0.139
F8	738	0.049	0.278	0.14
P7	723	0.021	0.329	0.142
P3	705	0.07	0.259	0.141
Pz	707	0.109	0.227	0.14
P4	684	0.125	0.219	0.141
P8	601	0.236	0.174	0.144

Provided *p*-values are unadjusted; no results survived FDR correction. SE effect size—standard error of effect size; and U—Mann–Whitney U test statistic.
